# On the External Use of the Nitrate of Lead

**Published:** 1853-01

**Authors:** 


*On the External Use of the Nitrate of Lead.* By Dr. Ogier Ward, Kensington.

Dr. Ogier Ward was induced to make use of this preparation in cases accompanied by fœtid discharges, observing its advantages as a disinfectant under other circumstances.

The first case in which he used it with this intention was that of a lady, whose lochia were so offensive as to scent the whole house, and nauseate even the nurse. It was used as an injection; and the third application effected a complete removal of the fœtor.

He has also used it with success as a lotion for sore legs when in a sloughy and indolent condition, and finds that it soon restores them to a healthy state, inducing a proper secretion of pus, with firm granulations on the surface of the ulcer.

He has not used the nitrate in acute gonorrhœa, but states that it acts admirably as an astringent in gleety discharges, as well as in those of cancer uteri, whether sanious or purulent. In short, as a lotion it is as extensively useful as the diacetate of lead, while it is superior to that preparation by its disinfecting property.

In chronic cutaneous diseases he has seen the most remarkable instance of its efficacy in a case of eruption, of a kind of rupia or impetigo of five years' standing. The complaint broke out on the vertex of a woman, æt. 50, and, leaving its original site, it has gradually crept down over the forehead, nose, and cheeks, to the level of the mouth. The primary form of the eruption consisted of inflamed flattened pustules, slightly elevated above the surrounding skin, which, discharging their contents, formed thick, rough, yellowish crusts, or scabs, fissured in all directions, like those of crusta lactea, which firmly adhering and growing from their base, like rupia, for a longer or shorter time, fell off at last, leaving cicatrices of various shapes, exactly like the seams and pits of small-pox. The patient came under Dr. Ward's care some months ago, when he tried many remedies, both internal and external, in vain, quinine being the only one that produced any good effect, and this not permanently. As the skin around the sores and where they had healed seemed in a state of hypertrophy, the papillæ projecting in many places as in elephantiasis, it occurred to him that the best way to check the progress of the disease would be to apply some penetrating astringent to the surface; and with this view he ordered a lotion of the nitrate of lead, with quinine internally. In the course of a few days the eruption ceased to make any progress, the crust began to fall off, and the skin to lose its redness and swelling; and in a fortnight every sore was healed, though the face and forehead remained still seamed with scars. There has been no fresh breaking out for some weeks, though, as the nose is still red and swelled, he has ordered the continuance of the lotion.

The formula he generally uses is the following:

℞ Plumbi Carb., scr. j;
Acid. Nitr. dil., q. s. ad solvendum;
Aquæ dist., ℔j.
M.

Fiat Lotio bis terve in die assidue utenda.—*Prov. Med. and Surg. Jour.*

